# Corrections

**Published:** 1860-04

**Authors:** 


### ARTICLE X.

### *Corrections.*

An amusing little piece has recently appeared, in "The Dental Register of the West," elegantly entitled "Another Screw Loose," to which we beg to direct the reader's attention. We, moreover, ask a moment's indulgence while we correct several errors into which the author of that piece has fallen in animadverting on an article on *Dental Societies*—which we had the honor to write for this Journal. Amusing as that performance certainly is in itself, we cannot avoid considering it in connection with similar efforts by the same author, and the aggregate of amusement thus available rather affects us to melancholy than to any other emotion. Our feelings corroborate one of the sayings of Pascal. He, although living before the rise of Dentistry, was yet able to excogitate the truth that "too much pleasure gives pain." Had he been born in this age, to witness the fantastic evolutions accomplished over our composition, it is impossible to conjecture the sublime heights of philosophy to which he would have arisen. The inimitable tricks of our harlequin critic, as he drags our remarks through the doubtful regions of his intellect until they are robbed of our meaning, would have given the great Frenchman ideas of amusement and pleasure to be sought for in vain at Port Royal or Paris. We, who have enjoyed these hitherto unknown advantages, are hence a most competent witness as to the truth of Pascal's saying. We have been delighted almost to suffering. We have laughed at the comedy but deplored the degradation of the player. We have examined "W's" polemical exacerbations, but to wonder at the infatuation which has led him to abandon his gold for his pen. How much good, according to his own notion of good, might he not accomplish by devoting his *whole* time to the production of that article by which he has acquired some reputation.

The criticisms of "W." suggest the idea of an Iroquois at battle, armed with an Australian boomerang. The ignorant, but vain-glorious savage, rushes on with triumphant whoops, but suddenly feels himself wounded, perhaps mortally, by his adopted instrument of death ; true, however, to the habits of his race, his dying gasp is a defiant, exultant cry, in the face of an uninjured enemy. So "W.," using unfamiliar arms, is continually worsted, but Iroquois like, he shouts with the air of a conqueror at every defeat. Judging from the past, "W.," in this case, will claim the old woman's privilege of "last word"—and this we cheerfully concede ; he shall enjoy his apparently much-prized practice of shooting the last arrow, for his aim is far too inaccurate and his mark too small for us to feel the least concern even though conscious of points open to attack.

Let us now proceed to refute "W's" strictures in the order in which they are made—assuring the reader before hand, that we shall not take advantage of the opportunity to reiterate any of the sentiments contained in *Dental Societies* further than the exigencies of the case may require. Our only object at this moment, is to prove the utter *incorrectness*, to use the mildest phrase, of every remark made by "W." in reference to our article.

His first stricture is summed up in the following remarkable interrogatory, which is evidently propounded in the expectation of forcing us to accept a dilemma, either horn of which would prove fatal to the cogency of the remarks under his consideration:—"Is a tree that monopolizes all the fruit of the orchard, bears all that is to be gathered, a failure?"—A child might answer the question, and yet, not be driven to the alternative that "W." intended, we trust that our readers will pardon us for answering so simple a question. The tree, though much diseased, may bear *some* badly developed, blighted fruit; the tree, diseased as it is, may bear *all* of the fruit in the orchard in which it stands—the other trees yielding actually no fruit at all. Now, is there any doubt that the imaginary orchard, including the tree, is a failure, or, that the tree, taken by itself, is a failure? Even in Cincinnati, where it is seemingly impossible for some persons to know whether a thing has or has not failed, the tree would be pronounced a *lamentable* failure. The experienced Pomologist—and we presume there are some in the West—would advise to have the *orchard* grubbed up, *the tree* included. Better use, he would say, could be made of the ground.

Our critic, our Iroquois we should have said, is now reveling in a garden of delight; another pomological inquiry succeeds the first. "But have local societies produced no fruit?" It is here implied that "fruit," *any* "fruit," has been denied to local societies. This, we have never done; but we have denied "true *scientific* fruit." And "W." has not shown us to be incorrect.

By way of controverting our remark as to the *two* creditable circumstances relating to the *national* societies, we are ingeniously informed of a host of the benefactions of a *local* society—the "Mississippi Valley Association." However influential or extensive, the "Mississippi Valley Association" carries the proof of its local character in its name; indeed it is *local* in every respect. Certainly none of the meritorious acts of that body—and we will admit them to be both important and dignified—can be urged as the result of scientific activity. Even "W." will not mention 'excavators,' 'socket rims,' 'lamps,' etc., as 'true scientific fruit.' They are useful, very useful; they indicate great ingenuity, but they cannot be offered as a vindication of *science* in dental societies.

"W." concludes this part of his subject as follows:—"And much might be said of the honorable and useful efforts of other local societies: but we must refrain. The above will show how little credit is to be given to such sweeping assertions as this writer seems fond of." It will be observed that, contrary to the practice of "W.," we give our quotations unmangled:—Here "W." in speaking well of the "honorable and useful efforts of other local societies," would imply that we have unjustly reprobated the institutions in which he, sagaciously, sees much good. So far is this from being the case, that while we denied that they had produced anything of scientific importance, which we pronounced the greatest good, we plainly approved of their establishment, when commenting upon that meeting of the convention which recommended them. Any credit, therefore, that "W." would appear to claim in view of discernment exhibited, in contra-distinction to our want of it, is wholly unwarrantable. It is well that he promises to "refrain." Instead of refraining, however, note the last clause of the sentence quoted. With the utmost effrontery we are charged with being fond of, or of *seeming* to be fond of, sweeping assertions. For once "W." is uncertain. Notwithstanding the fact "that the above will show how little credit is to be given to such sweeping assertions," he finishes the paragraph by stating that we only "*seem* to be fond of" those very things the "little credit" of which he shows by "the above." In other words, he proves that little credit is to be given for things which we practice, but he confesses that he does not know whether we have those things or not. Having incoherently attempted to convict us of using certain contradictory expressions, in the first part of his piece, the clever author of "Another Screw Loose," devises the expedient of "sweeping assertions" to strengthen his flimsy assaults. He supports his man of straw with a cobweb.

We next come to a long paragraph on the attributes of the old and the young *croaker*. Many surmises are made as to whether we are the one or the other. First, moral hints are aimed at us, we, meanwhile, sustaining the character of "old croaker." But fearing lest we should escape in that character, we are, with customary nimbleness, disposed of as "young croaker." Without divulging our incognito, however, we are glad to state that we could not strictly be assigned to either category. Nor have we ever been of the *profession*, or of the *auxiliary* branches of science, as understood by the American Dental Convention, though we have inadvertently made use of the word *our* in connection with the profession in some places. Hence we are not "disaffected" in the manner charged: nor have we taken the "back track," been "disappointed" or "confused." Moreover, we disclaim "martrydom," or the idea "that the only hope of the profession lies deep down in the recesses of our mind." We do not habitually "rebuke young men for their opinions," although this would not be amiss in some cases; nor do we tell old men "*experience.*" This last we unambiguously censured in *Dental Societies.* In short, we might affirm that we do *nothing* in the way and manner conjectured by "W." In his usually severe logical manner, he winds up his discussion on "croakers," by assuming the very point at issue, viz. that dental societies "have done more to accomplish the ends for which they were organized, than any other associations of the same period." But this is a trifle compared with the audacious trickery of the next paragraph, in which our critic attempts to show us up as affected by a "mathematical hallucination." "For example, in speaking of the number of essays read at the yearly meetings of the 'American Society,' he says: 'The gradual diminution in the number appointed and *read continued.*' Now let us examine this gradual *diminution*, which preceded the meeting he refers to. At the first meeting after the organization, according to his showing, 'not one was read.' By the next meeting they had *gradually diminished up to three,* and by the next, to 'three or *four.*'" Here "W." quotes the *last* four words of this clause: "but out of the twelve essays, the subjects of which had been publicly rehearsed at the preceding meeting and printed in large type, *not one was read.*" Now "W." must have been perfectly aware that the quotation of the *whole* clause would have prevented him from indulging in his humorous conceit of of the "mathematical hallucination." In continuation of that clause—in which it is plain that we say that none of those *appointed* were read—we state that in the place of the twelve appointed essays, *three* were offered on subjects which had *not* been named by the society.

From the foregoing it is hardly possible to avoid the alternative, that "W." is either the victim of most complicated misapprehensions, or he is the deliberate falsifyer of what he knew to be our meaning. Certainly the misapprehensions, if misapprehensions they are, bear strong marks of *design.* In either case, we have singularly enough, an apt illustration of "W's" own aphorism: "That men make themselves very ludicrous when determined to complain, having no just grounds." Our critic is not only determined to complain, but he also invents subjects of complaint—and they are of the smallest. No expression is sufficiently unimportant to escape his intellectual antennæ. Little crumbs of orthographical doubt supply fit pabulum for his diatomaceous digestion.

Next in order, we have a succinct, but complete, specimen of "W's" method of dealing with obnoxious remarks; here it is: "Of the two national societies, this writer tells us that 'their sole result has been to show that the right course is directly opposite to the one heretofore followed.' But it is plain that the two societies under consideration followed different courses—as nearly opposite as could well be conceived. To which one of these is the right course directly opposite." Now, without discussing the characteristics of the two societies, which are doubtless different in *many* respects it is only necessary to say that the *sole result* alluded to arose from the *freedom of access*, which we represented as a most pernicious influence. One of the great reforms we proposed was in opposition to this prominent element of the societies. Both societies permitted the evil; in doing so they followed the course directly opposite the right one. We are correct in thinking that the most saucy Grub street author would not have assailed us with such a miserable quibble as this.

The next point, if possible, is more strikingly absurd than any we have yet mentioned from "W's" pen. We give it in full. "But the old society having reached 'its full vigor,' we are told that it 'began rapidly to decline,' and that it 'tottered through two or three years of puerility and died.' Reached its full vigor and failed into *boyhood.*" This is but another instance of "W's" gross perversion of our language, and at the same time, a display of his own ignorance when he would appear wise. This last, however, we do not urge. We have chiefly to do with more positive sins. We never accused the society of having any (true) vigor at all; our words were that the society had reached "what we may *analogously* call its full vigor, and now began rapidly to decline." But "W." is not half satisfied with misquoting, he, over and above, gives us a new definition of a very common word. He should consult his dictionary before attempting such suicidal witticisms as the one on *puerility*. *Puerility* means *boyishness*, *childishness*, that which is trifling, not *boyhood*. An old man may totter through years of puerility. So may a society. Having thus ascertained the meaning of the word, "W." knows his condition. He has fallen, not into *boyhood*, but into peurility.

His logomachic labors over, our critic determines that we praised the second dental convention because we were present at that meeting, and at none of the others. This determination is entirely incorrect. We have never been present at any meeting of any dental society whatever. Our opinions have been formed from the printed records. Thus another inuendo as to the motives which actuated us, falls to the ground.

Finally: The *money test* receives the light of "W's" wisdom. In reference to this matter we have only to say that, while we do not for a moment suppose that he has impaired the soundness of our views, or damaged the testimony upon which they are based, we must protect ourselves from the supposition that we attempted to elaborate a *system*—as might be inferred from the tone of his remarks. On the contrary, we were careful to intimate that we only offered an outline. He appears, nevertheless, to feel some alarm when discussing the *five or ten minutes* rule which we had suggested, as one way at least of keeping idle persons silent. The fertility of his resources at length appear to be exhausted; for, far from seizing our evident meaning, he presumes to intimate that we intended this bare hint to have universal application. It is unnecessary to develop that hint here; but "W." should remember that, among intelligent men, special provision is always made for the reception of the words of the wise. We forbear, in conclusion, to say how the provision would operate on him in such an assemblage.

